# Supplementary material for: Modeling the impact of single-cell stochasticity and size control on the population growth rate in asymmetrically dividing cells
Source: PLoS Comput Biol. 2021 Jun 21;17(6):e1009080. doi: 10.1371/journal.pcbi.1009080 (PMC8248971; doi:10.1371/journal.pcbi.1009080)
Supplement: Attachment — Submitted filename: review.pdf [file pcbi.1009080.s009.pdf]

This is an interesting work addressing the impact of single-cell noises, growth morphology and cell divisions mechanisms, on the population growth rate of asymmetrically dividing cells. The authors recover that for symmetric divisions, a non-correlated noise on the individual growth rate leads to a decrease of the population growth rate, and further obtain that for a given level of that noise and for cells subject to strong size control, the population growth rate can increase as a result of introducing asymmetrical divisions. They also propose an explanation for the recent observation of positively correlated generation times in yeast, for certain size control policy for asymmetrically dividing cells. All these results are insightful and original, and are well illustrated by numerical simulations. I am thus favorable to publication, although the manuscript suffers a lack of clarity on certain points and some sections are ill-organized, which is why I ask that the authors carefully address the following points and make appropriate corrections :

- To explain their derivation of the Euler-Lotka equation for the two populations system in section 2.1 and in Suppl. Mat., the authors refer mainly to Ref 1, although the main idea seems to follow from Ref 12 by Powell. In Ref 2, the authors showed that the Euler-Lotka equation can be recovered from a simpler method than the one used by Powell, which is based on the consideration of the two sub-populations created by the first two daughter cells. This method is applicable here too. Although it may be less rigorous than the method used by Powell and may only apply to situations where there are no correlations, it is much simpler and it still has to work at least in that case, so it seems to me that it should be mentioned and discussed.
- In section 2.5, the authors attempt to explain why some important classes of cells divide symmetrically although they should in principle benefit from asymmetric divisions for their population growth rate given their size control policy and individual growth rate noise as shown in (Eqs. 5 and S43). The two proposed mechanisms are: non-exponential growth for extreme-sized cells, and correlated single cell growth rates. However, neither of these hypotheses is convincing. For the first mechanism (non exponential growth), the decrease of Λ_P when increasing x is notable only for weak size controls (fig S6 C,D), whereas Eqs. 5 and S43 hold precisely for strong size control. For strong size control, it can lead either to a decrease or not, depending on the values of ε and n (fig S6 A,B), so this is not a satisfactory argument For the second mechanism (correlated growth rate), the desired effect, that is a decrease of Λ_P when increasing x, that would explain why the majority of cells divide symmetrically, is only obtained for strongly correlated growth rates (PCC > 0.5). However, for the example they cite, E.Coli, PCC < 0.1, so it is in the range of values for which the population growth would benefit from asymmetrical division, but does not in experiments. This section lacks a conclusion to explain what the reader is supposed to make out of this and whether there are better arguments.
- The writing of Section 2.3, which is a key section in the article could be improved. The paragraph starts with a complicated equation in Eq 5, which is not discussed sufficiently nor tested numerically until after a rather long digression in line 130-143. In line 143, we finally get back to Eq. 5, but the main figure that supports it, namely fig S1B is not in the main text. This seems odd, given the importance of the point for that paper. In the next paragraph, the authors qualitatively test this behavior for other strategies of size control and claim that the threshold for a positive impact of x on Λ_P is $\alpha = 0.5$, although it does not appear clearly on fig 2B. Indeed, the adder curve seems to decrease with x, or at least be slightly impacted but not independent of it, and there is not much difference between the adder curve and the $\alpha = 0.1$ curve.
- In the same section, I did not understand well the implications of the difference between budding and non-budding cells in the theory because the figure 1C does not show well the difference graphically, and later in the paper, the difference in the simulation algorithm for these two mechanisms is not explained.

Minor points:

- On P3, I imagine $\tau_M < \tau_D$ should be replaced by the same inequality for averaged quantities.
- On page 9, the whole part on generation time correlations is related to section 9 of the SM, not section 8 as written on line 186. Also, it is also not very clear why a new function $h(V_b)$ needs to be introduced there instead of simply relying of that of Eq. 4, even if they match for the adder. On line 203, I guess fig S3A is meant.

- From Eq S49, it could be interesting to write down the PCC for $\alpha = 1$, showing that in this case PCC < 0 as mentioned in the main text (only qualitatively), even though it is still an increasing function of x. Correspondingly, on fig S3A, the fact the red curve remains below 0 can be highlighted.
- On fig 1A, the fact that the leaves should be included in the tree distribution could be better represented graphically, and explained in the caption.
- On line 168, for symmetrically dividing cells, noise in single cell growth rate can also increase the population growth rate, in presence of correlations.
- On fig 2C, σ_λ is not given.
- Page 15 lacks a conclusion to state that working out Eqs S58-60 gives the main result S49, as done below for sisters PCC.
- Inconsistency between Λ_p and Λ_P throughout the text.
- Line 347 'both both'.
