# Supplementary material for: Modeling the impact of single-cell stochasticity and size control on the population growth rate in asymmetrically dividing cells
Source: PLoS Comput Biol. 2021 Jun 21;17(6):e1009080. doi: 10.1371/journal.pcbi.1009080 (PMC8248971; doi:10.1371/journal.pcbi.1009080)
Supplement: Attachment — Submitted filename: Reviewer_responses_final.pdf [file pcbi.1009080.s010.pdf]

4/14/2021

Dear Editor,

Thank you for passing on the reviewers' comments on our submission "Modeling the impact of single-cell stochasticity and size control on the population growth rate in asymmetrically dividing cells". We have addressed the reviewers' feedback in our updated manuscript, and have discussed each reviewer's comments below with reference to the updated text. We have highlighted the changes made in response to the reviewers comments.

Best wishes,

Felix Barber, Jiseon Min, Andrew W. Murray and Ariel Amir

Reviewer #1:

> *This is an interesting work addressing the impact of single-cell noises, growth morphology and cell divisions mechanisms, on the population growth rate of asymmetrically dividing cells. The authors recover that for symmetric divisions, a non-correlated noise on the individual growth rate leads to a decrease of the population growth rate, and further obtain that for a given level of that noise and for cells subject to strong size control, the population growth rate can increase as a result of introducing asymmetrical divisions. They also propose an explanation for the recent observation of positively correlated generation times in yeast, for certain size control policy for asymmetrically dividing cells. All these results are insightful and original, and are well illustrated by numerical simulations. I am thus favorable to publication, although the manuscript suffers a lack of clarity on certain points and some sections are ill-organized, which is why I ask that the authors carefully address the following points and make appropriate corrections:*

We thank reviewer 1 for their encouraging feedback on our submission.

- *To explain their derivation of the Euler-Lotka equation for the two populations system in section 2.1 and in Suppl. Mat., the authors refer mainly to Ref 1, although the main idea seems to follow from Ref 12 by Powell. In Ref 2, the authors showed that the Euler-Lotka equation can be recovered from a simpler method than the one used by Powell, which is based on the consideration of the two sub-populations created by the first two daughter cells. This method is applicable here too. Although it may be less rigorous than the method used by Powell and may only apply to situations where there are no correlations, it is much simpler and it still has to work at least in that case, so it seems to me that it should be mentioned and discussed.*

Thank you for pointing this out. As you noted, the method used by Powell and the simpler approach used in Ref. 2 both assume no correlations. We have included a description of the simpler approach in the main text (lines 108-114) to assist the reader. We have also added text to emphasize both the connections with and distinctions from the work of Powell in the supplementary text, copied below.

Main text:

"In the case with no generation time correlations, Equation 3 can be derived simply in a similar manner to that described in [2] by noting that a population seeded from a single cell that divides at time 0 will grow exponentially as $e^{\Lambda^P t}$, which must match the size of the two populations seeded by the two progeny cells, with combined sizes growing as $2\int f^P{}_0(\tau)e^{\Lambda^P(t-\tau)}d\tau$. Dividing through by $e^{\Lambda^P t}$ then yields the desired result. Section S2 details our derivation of Equation 3 for the case of correlated generation times, similar to a prior approach that addressed symmetrically dividing cells [2]."

SI:

"In doing so we follow a similar approach to prior work [1, 2]. This approach extends early work by Powell to consider the case of correlated generation times in asymmetrically dividing populations [2]."

- *In section 2.5, the authors attempt to explain why some important classes of cells divide symmetrically although they should in principle benefit from asymmetric divisions for their population growth rate given their size control policy and individual growth rate noise as shown in (Eqs. 5 and S43). The two proposed mechanisms are: non-exponential growth for extreme-sized cells, and correlated single cell growth rates. However, neither of these hypotheses is convincing. For the first mechanism (non exponential growth), the decrease of ΛP when increasing x is notable only for weak size controls (fig S6 C,D), whereas Eqs. 5 and S43 hold precisely for strong size control. For strong size control, it can lead either to a decrease or not, depending on the values of ε and n (fig S6 A,B), so this is not a satisfactory argument For the second mechanism (correlated growth rate), the desired effect, that is a decrease of ΛP when increasing x, that would explain why the majority of cells divide symmetrically, is only obtained for strongly correlated growth rates (PCC>0.5). However, for the example they cite, E.Coli, PCC < 0.1, so it is in the range of values for which the population growth would benefit from asymmetrical division, but does not in experiments. This section lacks a conclusion to explain what the reader is supposed to make out of this and whether there are better arguments.*

We thank the reviewer for pointing this out. This inconsistency between our expectations based on the exponential growth rate results and the experimentally observed behavior is considered at length in the discussion section, but we have added relevant text to section 2.5 as well.

"Our findings show that biologically relevant growth rate correlations are unlikely to be large enough to significantly alter the growth rate gains associated with asymmetric division. Further, Figure S6 only showed a substantial effect of a growth rate penalty on cells with weak strategies of cell size control. This leaves open the question of why symmetrically dividing cells are so prolific, when dividing asymmetrically with a strong size control strategy can in theory enhance the population growth rate. This discrepancy between our predictions and experimental observations may arise from the fact that the exponential growth rate is not the only physiological variable that is likely to be evolutionarily selected for. Alternatively, it may indicate that cells are unable to tune their division asymmetry without causing other adverse physiological effects, preventing them from exploiting the growth rate gains associated with asymmetric division. We expand on these points further in the discussion."

- *The writing of Section 2.3, which is a key section in the article could be improved. The paragraph starts with a complicated equation in Eq 5, which is not discussed sufficiently nor tested numerically until after a rather long digression in line 130-143. In line 143, we finally get back to Eq. 5, but the main figure that supports it, namely fig S1B is not in the main text. This seems odd, given the importance of the point for that paper. In the next paragraph, the authors qualitatively test this behavior for other strategies of size control and claim that the threshold for a positive impact of x on ΛP is $\alpha = 0.5$, although it does not appear clearly on fig*

2B. Indeed, the adder curve seems to decrease with x, or at least be slightly impacted but not independent of it, and there is not much difference between the adder curve and the α = 0.1 curve.

We thank the reviewer for pointing this out. We have amended Figure 2 in the main text accordingly. We have also rewritten parts of the discussion of Equation 5 in the main text, as well as rewriting the description of the impact of cell size control strategy on the growth-rate gain for increased clarity. Various changes are highlighted between lines 179-228.

- *In the same section, I did not understand well the implications of the difference between budding and non-budding cells in the theory because the figure 1C does not show well the difference graphically, and later in the paper, the difference in the simulation algorithm for these two mechanisms is not explained.*

We are sorry for the confusion on this point. We have updated Figure 1 to better illustrate the distinction between budding and non-budding cell types, and have added corresponding text both in the figure legend and in the main text to better describe this point.

"The min and max conditions for budding cell size at birth are related to the observation that in this case, mother cells will only ever increase in size over subsequent cell cycles (see methods). In the expression for volume at division V_d, η represents time-additive noise in the timing of cell division."

"Budding cells establish the plane of division early in the cell cycle at the boundary of the cell, and from then on direct further growth towards a newly forming bud on the other side of the division plane. This has the consequence that the volume of mother cells will only ever increase when tracked over multiple generations, whereas for non-budding cells, a mother cell may be born smaller than its parent cell was at birth. In general, large mother cells may arise whose volume at birth represents a greater fraction of their projected volume at division than would be allowed by the division asymmetry we impose. To address this case, and to ensure that cell size only increases with each generation in our simulations, our model for a budding morphology applies the condition that $V_b^{n+1}=\max(V_b^n,(1+x)V_d^n/2)$. In this case we assume that the division timing still follows Equation 4. This condition implicitly assumes that these large cells will begin budding immediately upon birth, producing daughter cells that represent a smaller fraction of the mother cell size than would be expected based on the division asymmetry we typically impose. In fact, without time-additive noise this ``maximum'' condition for budding cells is never invoked, since $\max(V_b^n,(1+x)V_d^n/2)$ consistently favors the second term. This can be readily seen, since by following the growth policy of Equation 4 with a given division asymmetry x, a mother cell's size at birth will increase monotonically over successive generations (i.e. $V_b^{n+1}=(1+x)V_d^n/2>V_b^n$) towards a fixed point at $V_b^*=\alpha\Delta(1+x)/(\alpha(1+x)-x)$. Cells born at this fixed point will begin budding immediately, and will produce daughter cells whose size is consistent with the division asymmetry we impose. A sizer model without time-additive noise represents a special case of this, wherein all mother cells will begin budding immediately. Since the ``maximum'' condition is never invoked when time additive noise is zero, budding cells will grow identically to non-budding cells in this case, as demonstrated in Figure S1 (A) for $\sigma_t=0$. Consequently, within our implementation of budding, our analytical results for the population growth rate without noise in generation times are consistent for both budding and non-budding morphologies. In the presence of generation time noise this equality need not hold; however, the effect of growth morphology on the population growth rate remains minimal in this case as seen in Figure S1 (A)

for σ_t >0. We therefore concluded that the choice of budding vs. non-budding growth morphologies did not impact the population growth rate substantially, and all results presented herein are valid for both budding and non-budding morphologies unless explicitly stated otherwise. For our investigations of generation time correlations, the general conclusions are also independent of growth morphology, but for completeness we present simulated results for both growth morphologies to highlight the relevant differences."

Minor points:

- *On P3, I imagine τM < τD should be replaced by the same inequality for averaged quantities.*

In the study this section refers to, τ_M and τ_D are held to be constant as described in the text. We have updated the text to clarify this point as follows:

"One early study in budding yeast theoretically predicted the dependence of Λ_P on the division times for daughters τ_D and for mothers τ_M, with τ_M and τ_D assumed to be constant (Hartwell, 1977) (Section S3)."

- *On page 9, the whole part on generation time correlations is related to section 9 of the SM, not section 8 as written on line 186. Also, it is also not very clear why a new function h(Vb) needs to be introduced there instead of simply relying of that of Eq. 4, even if they match for the adder. On line 203, I guess fig S3A is meant.*

We thank the reviewer for pointing this out, and have updated the relevant references. The updated function for the volume at birth in Eq. 7 was adopted for mathematical convenience, and we have added text to explain this:

"We adopted this expression to assist in making analytical predictions for the generation time correlations."

- *From Eq S49, it could be interesting to write down the PCC for α = 1, showing that in this case PCC < 0 as mentioned in the main text (only qualitatively), even though it is still an increasing function of x. Correspondingly, on fig S3A, the fact the red curve remains below 0 can be highlighted.*

We agree it is an interesting case, and have added the specific functional form in Equation S50, as well as adding text in the legend of Figure S3A to note this fact.

"Note that for α=1, the PCC remains fixed below zero as predicted by Equation S50."

- *On fig 1A, the fact that the leaves should be included in the tree distribution could be better represented graphically, and explained in the caption.*

We thank the reviewer for their suggestion, and have addressed this in the figure.

- *On line 168, for symmetrically dividing cells, noise in single cell growth rate can also increase the population growth rate, in presence of correlations.*

We have added text to address this point:

“We will show in Section 2.5 that the population growth rate may in theory be enhanced by introducing strong single cell growth rate correlations (for both symmetrically and asymmetrically dividing cells), however, this effect is distinct from the one discussed here.”

- *On fig 2C, $\sigma\lambda$ is not given.*

This has been updated accordingly.

- *Page 15 lacks a conclusion to state that working out Eqs S58-60 gives the main result S49, as done below for sisters PCC.*

This has been updated accordingly.

- *Inconsistency between Λp and ΛP throughout the text.*

This has been updated to maintain consistency.

- *Line 347 ‘both both’.*

This has been updated accordingly.

Reviewer #2:

This is a comprehensive theoretical study linking the population growth rate to size control strategies and the various single cell level stochasticity in asymmetrically dividing microbial cells, extending their study on symmetrically dividing ones. The results are valuable and insightful, and can hopefully motivate further studies on some real examples (such as the budding yeast). I have only a few minor points:

We thank the reviewer for their positive views on our manuscript.

(1) The author used h(v_b) in the text (e.g. line 98) but f (v_b) in Figure 1.

We thank the reviewer for pointing this out. It has been updated accordingly.

(2) Line 347: both both.

This has been updated accordingly.

(3) In several places in the text, the author used the budding yeast as examples. But their study, as they pointed out in line 111-118, is essentially entirely on the non-budding case (Figure 1B) (BTW, it would help to list some real examples of the non-budding case.) This may cause some confusion.

We have added additional text to clarify that these growth morphological differences of budding vs. non-budding have no impact when time additive noise is zero, and have minimal impact on the population growth rate otherwise. We have also mentioned the example of *Caulobacter crescentus* and the Mycobacterium genus, as examples of asymmetrically dividing, non-budding bacterium.

"The most prevalent example of an asymmetrically dividing, budding cell is *S. cerevisiae*, while asymmetrically dividing, non-budding cells include the Gram-negative bacterium *Caulobacter crescentus*, as well as various asymmetrically dividing mycobacteria (Hartwell 1977, Skerker 2004, Logsdon 2018)."

"To address this case, and to ensure that cell size only increases with each generation in our simulations, our model for a budding morphology applies the condition that $V_b^{n+1}=\max(V_b^n,(1+x)V_d^n/2)$." … "Since the ``maximum'' condition is never invoked when time additive noise is zero, budding cells will grow identically to non-budding cells in this case, as demonstrated in Figure S1 (A) for $\sigma_t=0$. Consequently, within our implementation of budding, our analytical results for the population growth rate without noise in generation times are consistent for both budding and non-budding morphologies. In the presence of generation time noise this equality need not hold, however, the effect of growth morphology on the population growth rate remains minimal in this case as seen in Figure S1 (A) for $\sigma_t>0$. We therefore concluded that the choice of budding vs. non-budding growth morphologies did not impact the population growth rate substantially, and all results presented herein are valid for both budding and non-budding morphologies unless explicitly stated otherwise. For our investigations of

generation time correlations, the general conclusions are also independent of growth morphology, but for completeness we present simulated results for both growth morphologies to highlight the relevant differences.”

Reviewer #3:

This article studies the impact of size control mechanism and the single-cell randomness on the population growth rate in asymmetrically dividing cells. This work is closely related to the previous work (reference [2]) by the correspondance author. The major difference is that [2] was based on symmetrically dividing cells while this manuscript is about asymmetrically dividing cells. One important result from this work is the conclusion that noise in the single-cell growth rate enhance the population growth rate for cells with strong size control. This is different from the case in symmetrically dividing cells, in which the noise in single-cell growth rate decrease the population growth.

Overall, this manuscript presented a fairly complete work with solid mathematical analysis and computer simulation based on simple models of cell size control. Both the results and the discussion are very interesting.

We thank the reviewer for their positive assessment of our work.

One drawback of this work probably is the lack of direct support of wet-lab experiments. Although there were experimental results that the authors' analysis could match with, they are not direct support. There are mutant cells that are viable in certain medium even without size control. I think the corresponding correlation data can be used to verify the validity of the analysis. I suggest that the authors make a few prediction based on possible experimental setting, so that the community could proceed and verify (or deny) the results presented with this analysis.

We thank the reviewer for this helpful suggestion. It is true that while there is experimental support for positive generation time correlations in asymmetrically dividing cells, this support is lacking for our conclusions regarding the population growth rate. To address this, we have included the following text in the discussion section.

"One clear modeling prediction presented herein is that for cells with strong size control, increasing the division asymmetry while not otherwise perturbing cell physiology should cause the population growth rate to increase. Experimental tests for this could include perturbing the proteins responsible for establishing the cell midpoint as the point of contractile ring formation in rod-shaped cells, such as by perturbing Pom1 function in the fission yeast *Schizosaccharomyces pombe* (Moseley, 2009) or the Min system in *E. coli* (Rowlett, 2013). The prediction in either case would be that deviations from symmetric division will increase the population growth rate, provided the single cell growth rate distributions remain unchanged. Our work also highlights a clear need to assess the effect of cell size perturbations on cell growth rate. Experimental work in this area would assess whether a growth rate penalty for extremely large or small cells may function to limit the growth-rate gains associated with asymmetric division. A related, testable hypothesis is that asymmetrically dividing cells in nature exist at a local maximum in Λ_P resulting from a balance between the aforementioned growth rate gains of asymmetric division and a growth rate penalty for unusually sized cells (Figure S6). If this hypothesis is true, our model makes the intuitive, experimental prediction that weakening size control substantially in asymmetrically dividing cells without adversely affecting other physiological parameters will lead to a decrease in the population growth rate. This could in principle be done by perturbing the function of molecular regulators of cell size, however, the

identities of these cell size control mediators remain largely unknown, meaning that perturbations to the spread in cell size may be confounded by accompanying perturbations to the average cell size."

> *The other problem is related to the size control mechanism used in the analysis. I'm particularly concerned with the simple model of size control, as it is the basis of the whole analysis but it lacks of necessary discussion. For the budding yeast cells, the size control applies mainly to the G1 phase. Wet lab experiments show a clear negative correlation between the birth volume and time in the G1 phase for daughter cells with smaller sizes, but the corresponding correlation for mother cells and daughter cells with larger sizes are much lower. In the manuscript the authors are aware of the difference of cell size control mechanism for mother and daughter cells, as it was discussed a little bit in the Discussion section. I wonder how the correlation for the small volume and the time in G1 phase, rather than a whole cell cycle time is represented here. If the cell size control mechanism cannot be simply represented by equation (4) on page 5, then will all the analysis afterwards apply? Or what kind of changes will it lead to? I think this is a fundamental question this manuscript did not answer and it definitely worth some analysis and discussion.*
>
> *I think these two questions I raised here are challenging and may be hard for the authors to provide immediate and complete answer. I expect the authors to give sufficient discussion on them, though.*

We thank the reviewer for their helpful comments. We agree that Equation 4 simplifies matters by considering the whole cell cycle rather than breaking the cell cycle into individual phases. However, an adder model (α=1/2) has been demonstrated in the past to provide an effective fit to various cell cycle parameters, including the dependence of cell cycle duration on cell size in budding yeast daughter cells. To demonstrate this, we have included relevant plots from a previously published work (Soifer *et al.*, 2016) here.

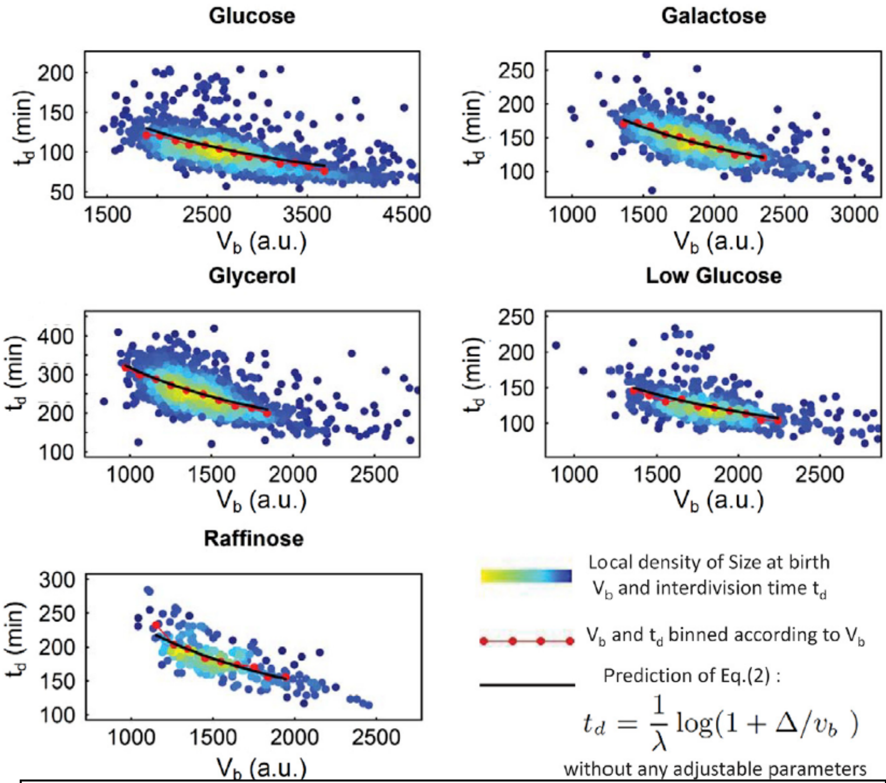

Figure 2 from Soifer *et al.*, 2016. showing behavior consistent with an adder model in budding yeast (equivalent to Eq. 4 from our submission with α=1/2), with no fitting parameters.

As a further control for the effects of more specific strategies of cell cycle control, Section S5 and Figure S1C both address the effect of introducing a more "mechanistic" inhibitor dilution model that

breaks the cell cycle down into the pre-Start G1 phase and the post-Start budded phase. This abstracted molecular model has been shown in the past to provide an effective fit to experimental data (see for example our previous work Barber *et al.* 2017, as well as Soifer *et al.* 2016). We have copied the relevant text from the manuscript below:

"To first ensure that our results were robust to minor differences in model structure, we simulated cells following a more detailed inhibitor dilution model. In this model, a stable molecular inhibitor of cell cycle progression must be diluted through growth in order for cells to pass through an essential cell cycle checkpoint (known as Start in the case of budding yeast), and is then newly synthesized prior to cell division. Our prior work investigated the adder correlations that arise within an inhibitor dilution model (Barber et al., 2017). Here we use a tunable parameter *a* to describe the degradation of some fraction of a cell's stock of inhibitor once the cell has passed through Start. By tuning *a* between 0 and 1, this can vary the strategy of size control between a sizer at *a=1* in which all a cell's inhibitor is completely degraded and newly synthesized with each cell cycle, and an adder at *a=0* in which no degradation takes place but inhibitor synthesis still occurs (see Section S5 for details). The sizer case of this model with a=1 shows good agreement with Equation 5 (see Figure S1 (C)), while the qualitative behavior of decreasing growth rate for weaker size control strategies reproduces that obtained using Equation 4. We also tested whether this inhibitor dilution model was able to generate robust positive correlations between the generation times of closely related cells, finding that despite quantitative differences arising from differences in model structure, the qualitative findings of Section 2.4 remain intact in this case, as shown in Figure S4 (E,F)."

We believe that since the models we employ are based on experimental evidence in previously published work, this treatment adequately addresses the first part of the author's question. We have added text to clarify that an adder model provides an effective description of cell size control in budding yeast daughter cells as follows:

"Prior work has shown that an adder model with $\alpha=1/2$ effectively captures the size-dependence of cell cycle progression in diploid, daughter budding yeast cells, indicating that Equation 4 is adequate as a generic model of cell size control (Soifer et al., 2016)."

To address the differences in cell size control between daughter and mother cells, one could allow for differences in the size control strategy adopted by these two cell types. Experimental evidence suggests that the slope of a linear regression between volume at division and volume at birth in budding yeast mother cells is between 1.1-1.3 across 5 growth media (Soifer et al., 2016). This slight difference from the slope of 1 predicted by an adder model and measured in daughter cells is unlikely to have a significant effect on the population growth rate. One can estimate the size of such an effect by considering the behavior our model predicts for such a slope (corresponding to roughly $\alpha\sim0.35$-0.45). Figure 2C demonstrates that within this regime of size control strategies weaker than an adder, there is little dependence of the relationship between Λ_P and division asymmetry on α, indicating that considering multiple strategies of cell size control for different cell types is unlikely to change our predictions for the biologically relevant regimes. We can see again that our conclusions regarding positive generation time correlations in Figure S3 (A) and S4 (A), or regarding the potential effects of a growth rate penalty model in Figure S5 are qualitatively unchanged by a slight weakening of the strategy of size control. We have included the following text to explain this argument:

“We believe this simplification is reasonable, since experimental evidence suggests that the slope of a linear regression between volume at division and volume at birth in budding yeast mother cells is between 1.1-1.3 across 5 growth media (Soifer et al., 2016). This deviation from the slope of 1 predicted by an adder model and measured in daughter cells is unlikely to have a significant effect on the population growth rate. We can estimate the magnitude of such an effect by considering the behavior our model predicts if this weaker strategy of size control were applied to the full cell population (corresponding to roughly $\alpha \sim 0.35$-0.45). Figure 2C demonstrates that within this regime of size control strategies weaker than an adder, there is little dependence of the relationship between Λ_P and division asymmetry on α, indicating that implementing multiple strategies of cell size control for different cell types is unlikely to change our predictions for the biologically relevant regimes. Furthermore, our predictions for positive generation time correlations in Figure S3 (A) and S4 (A), or regarding the potential effects of a growth rate penalty model in Figure S5 remain qualitatively unchanged if we expand the regimes that we consider to be biologically relevant to budding yeast to include slightly weaker strategies of size control.”

We have also added clarifying text to the same paragraph in the discussion that considers the effect of extremely rapid cell divisions in extremely asymmetric divisions for cells with strong strategies of size control:

“This assumption is expected to break down once larger mother cells divide rapidly enough to be limited by the replication and segregation of chromosomes, but this regime is not explored biologically in any of the asymmetrically dividing cell types we are aware of.”
